# Inhibition of Pyruvate Kinase M2 Markedly Reduces Chemoresistance of Advanced Bladder Cancer to Cisplatin

**DOI:** 10.1038/srep45983

**Published:** 2017-04-05

**Authors:** Xing Wang, Fenglin Zhang, Xue-Ru Wu

**Affiliations:** 1 Department of Urology, New York University School of Medicine, New York, NY 10016, USA; 2 Department of Pathology, New York University School of Medicine, New York, NY 10016, USA; 3 Veterans Affairs New York Harbor Healthcare System Manhattan Campus, New York, NY 10010, USA.

## Abstract

Chemoresistance to cisplatin is a principal cause of treatment failure and mortality of advanced bladder cancer (BC). The underlying mechanisms remain unclear, which hinders the development of preventive strategies. Recent data indicate that pyruvate kinase M2 (PKM2), a glycolytic enzyme for Warburg effect, is strongly upregulated in BC. This study explores the role of PKM2 in chemoresistance and whether inhibiting PKM2 augments the chemosensitivity to cisplatin and reduces BC growth and progression. We found that Shikonin binds PKM2 and inhibits BC cell survival in a dose-dependent but pyruvate kinase activity-independent manner. Down-regulation of PKM2 by shRNA blunts cellular responses to shikonin but enhances the responses to cisplatin. Shikonin and cisplatin together exhibit significantly greater inhibition of proliferation and apoptosis than when used alone. Induced cisplatin-resistance is strongly associated with PKM2 overexpression, and cisplatin-resistant cells respond sensitively to shikonin. In syngeneic mice, shikonin and cisplatin together, but not as single-agents, markedly reduces BC growth and metastasis. Based on these data, we conclude that PKM2 overexpression is a key mechanism of chemoresistance of advanced BC to cisplatin. Inhibition of PKM2 via RNAi or chemical inhibitors may be a highly effective approach to overcome chemoresistance and improve the outcome of advanced BC.

Bladder cancer (BC) or urothelial carcinoma of the bladder is the fourth most prevalent cancer in men and the costliest cancer to manage^1^,^2^. While low-grade and early-stage tumors in general have a favorable prognosis, advanced BC is among the most aggressive cancers with high morbidity and mortality^3^,^4^. According to the American Cancer Society, the 5-year survival rate for regionally and distantly metastatic BC is about 34 and 5%, respectively^1^. Despite intense efforts over the past four decades treatment options remain scant. The mainstay of treatment for advanced BC is cisplatin-based neoadjuvant therapy prior to radical cystectomy for muscle-invasive BC and cisplatin as a single agent or as a key component in combination chemotherapy (such as MVAC) for metastatic BC^4^. However, preexisting chemoresistance is encountered in a sizable portion of the patients, particularly in recently recognized “p53-like” variant of the luminal subtype and certain basal-subtype muscle-invasive BC^5^,^6^,^7^. For those who show initial response, resistance eventually emerges in a majority of the cases, resulting in treatment failure and disease progression^8^. Recent clinical trial studies exploiting immune-checkpoint blockade with monoclonal antibodies against PD-1 and PD-L1 for metastatic BC have shown highly encouraging results, although only one-fifth of the patients who overexpress PD-1 and/or PD-L1 will likely benefit^4^,^9^. Clearly, there is a pressing need to explore additional avenues to more effectively treat advanced BC as a whole.

Pyruvate kinase is an enzyme that functions in the glycolytic pathway and catalyzes the last, rate-limiting step of glycolysis by converting phosphoenolpyruvate and ADP to pyruvate and ATP^10^,^11^. Of the four known isoforms, the muscle-type pyruvate kinase (PKM) gene is expressed ubiquitously and capable of producing two mRNA products through alternative use of exon 9 (PKM1) or exon 10 (PKM2)^12^. While normally PKM1 is present in adult cells, PKM2 is expressed abundantly in embryogenic tissues. During tumorigenesis, however, a major isoform switch occurs that replaces PKM1 with PKM2. The latter isoform is in fact associated with a reduced pyruvate kinase activity, leading to the accumulation of intermediate products that are necessary for tumor cell biosynthesis of amino acids, lipids and nucleic acids^10^,^11^,^13^. In addition to altering the tumor cell metabolism, PKM2 has been shown to exert direct oncogenic effects in part by acting as a protein kinase and interacting with growth-promoting proteins such as beta-catenin, STAT3, FGFR1, A-Raf and PKC^13^,^14^; increasing the transcription of cell-cycle drivers such as cyclin D1 and hypoxia-related genes such as HIF1^15^; and remodeling the histones^14^. Not surprisingly, downregulation of PKM2 by specific inhibitory RNAs could effectively decrease cell viability, increase apoptosis *in vitro* and inhibit the growth of xenografted tumors^16^. Targeting PKM2 through chemical inhibitors has also been explored recently. Of particular interest was the finding that shikonin, an active compound found in medicinal plants *Lithospermum erythrorhizon*, binds PKM2 during affinity purification^17^ and strongly inhibits cell proliferation and induces apoptosis in non-BCs^14^,^18^.

While the role of PKM2 in bladder tumorigenesis and whether inhibiting PKM2 affects BC growth and progression remain unclear, two recent developments are worth noting. Using an unbiased proteomic profiling we found PKM2 to be a principally upregulated protein during urothelial tumor formation in low-grade non-invasive pathway of BC in transgenic mice expressing an activated HRAS^19^. We subsequently extended this finding into human BC cell lines and tumors, establishing PKM2 overexpression in both low-grade non-invasive and high-grade invasive human BC^19^. We also showed co-localization of PKM2 with BC progenitor cell markers, such as cytokeratins 5 and 14, suggesting a role of PKM2 in driving invasive bladder tumorigenesis. Finally by down-regulating PKM2 with specific siRNA and shRNA we showed that PKM2 was necessary for BC cell proliferation and survival^19^. The second development was the recent data from RNA-seq of The Cancer Genome Atlas (TCGA) network. The group found 97% PKM2 transcripts and only 3% PKM1 transcripts in 131 cases of human muscle-invasive BC profiled^20^. Thus, PKM2 is strongly overrepresented in not only mouse but also human muscle-invasive BC, and inhibition of PKM2 may be an attractive therapeutic approach for advanced BC. The present study was designed to test this idea by inhibiting PKM2 alone or in combination with conventional chemotherapeutic approach using *in vitro* and *in vivo* models.

## Results

### Shikonin Binds PKM2 and Inhibits BC Cell Survival at a Concentration Not Significantly Affecting the Protein’s Pyruvate Kinase Activity

**Figure 1:**
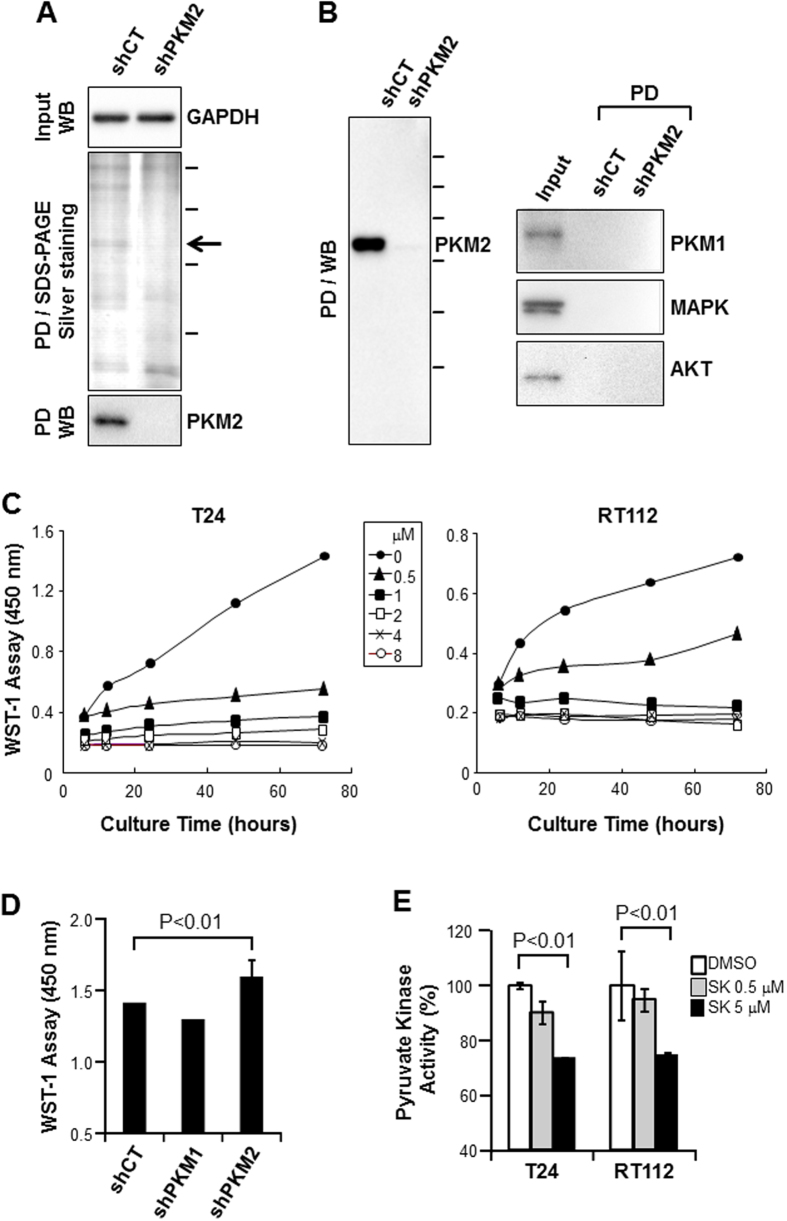
Shikonin binds PKM2 and inhibits BC cell proliferation independent of its enzymatic activity. (**A** and **B**) Total proteins extracted from BC cell line T24 that was stably transduced with control shRNA (shCT) or with shRNA of PKM2 (shPKM2) were incubated with solid-phase shikonin. After pull-down (PD), bound proteins were eluted with SDS-sample buffer, resolved on SDS-PAGE and stained with silver nitrate or immuno-blotted with various antibodies as indicated. Note, upon Western blotting (WB) of GAPDH, the equal amounts of input total proteins in the shCT cells and shPKM2 cells (**A**, top panel); the presence of a 55-kDa protein species (**A**, middle panel; arrow) only in the shCT cells; and the strong reactivity of this protein with anti-PKM2 antibody on Western blotting (lower panel). The result was reproducible in another pull-down experiment followed by Western blotting (**B**) showing the pull-down of PKM2, but not PKM1, MAPK or AKT, by solid-phase shikonin. Some gel and blot images were cropped to save space and their full-length versions are available upon request. (**C**) BC cell lines T24 and RT112 were incubated in the culture media containing various concentrations of shikonin and their proliferation status was assessed by WST-1 assay at the time points indicated. Note the dose- and time-dependent inhibition of cell proliferation by shikonin. (**D**) T24 cells stably transduced with shCT, shPKM1 and shPKM2 were incubated with the culture media containing 0.5 μM shikonin for 24 hours and assayed by WST-1. Note that the down-regulation of PKM2, but not that of PKM1, blunted the inhibitory effect of shikonin. (**E**) Cultured T24 and RT112 cells were treated with DMSO, 0.5 μM shikonin or 5 μM shikonin and the equal amounts of cell lysates were subject to pyruvate kinase activity assay. Note that, at the concentration that markedly inhibited cell proliferation (0.5 μM; panel **C**), shikonin did not significantly affect the pyruvate kinase activity (**E**).

To verify whether the binding between shikonin and PKM2, a phenomenon recently observed in non-BC cells, was operative in BC cells, we adopted a previously described pull-down procedure by incubating total protein extracts from T24 BC cell line with solid-phase shikonin^17^. With the equal amounts of total protein input as illustrated by Western blotting of GAPDH (Fig. 1A, upper panel), shikonin pulled down, upon SDS-PAGE and silver-nitrate staining, a 55-kDa protein species from T24 cells stably expressing a non-specific, control shRNA (shCT), but not from T24 cells stably expressing an shRNA of PKM2 (shPKM2) (Fig. 1A, middle panel). Western blotting using anti-PKM2 antibody established that the 55-kDa band was PKM2. An independent pull-down experiment reproduced the results (Fig. 1B, left panel), and further showed that shikonin did not pull down PKM1, MAPK or AKT, even though these proteins were present in the protein input (Fig. 1B, right panel). These results add further support to the recently demonstrated interaction between shikonin and PKM2^17^, and suggest that PKM2 could be a key target of shikonin in BC cells.

We next examined whether shikinon was inhibitory of BC cell proliferation and, if so, the basic parameters of such inhibition. Two cell lines, T24 and RT112, both derived from high-grade human BC, were incubated with various concentrations of shikonin and assayed for cell proliferation at different time points (Fig. 1C). A dose-dependent and relatively time-independent inhibition was observed, with significant inhibition achieved at a concentration as low as 0.5  μM for both cell lines. This inhibitory effect of shikonin on BC cells was at least in part PKM2-specific because PKM2 knockdown, but not that of isoform PKM1, significantly blunted the inhibition of shikonin on cell proliferation (Fig. 1D). Furthermore, at the concentration of cell proliferation inhibition, e.g., 0.5 μM, shikonin did not significantly impair pyruvate kinase activity in both cell lines tested (Fig. 1E), suggesting that inhibition of the oncogenic effects of PKM2 by shikonin was largely independent from its effects on glycolysis.

### Inhibition of PKM2 Potentiates the Responses of BC Cells to Cisplatin

**Figure 2:**
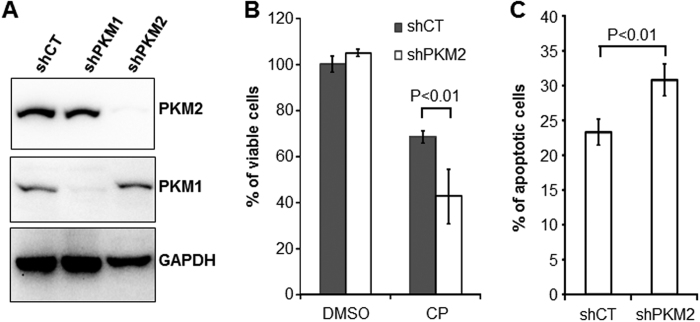
Down-regulation of PKM2 enhances the sensitivity of BC cells to cisplatin. (**A**) shRNA knockdown of PKM2. Western blotting of T24 cells with stable knockdown of PKM2, control (sh-CT) and PKM1 (sh-PKM1) showing the specific down-regulation of PKM2 without affecting PKM1. The blot images were cropped to save space and their full-length versions are available upon request. (**B** and **C**) T24 cells stably transduced with shCT and shPKM2 were treated with 1 μg/ml cisplatin for 72 hours and subjected to either WST-1 assay (**B**) or fluorescence-activated cell sorting after Annexin V staining (**C**). Note that cisplatin decreased proliferation and increased apoptosis of T24 cells in which PKM2 was knocked down, compared to T24 cells without PKM2 knockdown.

**Figure 3:**
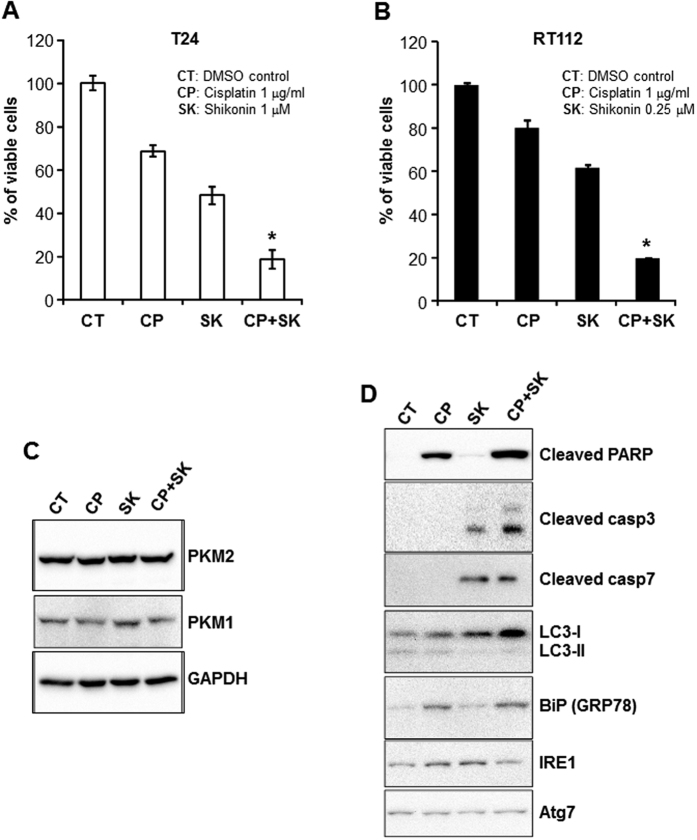
Combined effects of shikonin and cisplatin on BC cells. (**A**) T24 cells were incubated with culture media containing DMSO as control (CT), 1 μg/ml cisplatin (CP), 1 μM shikonin (SK) or both for 72 hours and then subjected to WST-1 cell proliferation assay. (**B**) RT112 cells were treated similarly with the exception of shikonin concentration used at 0.25 μM. Note the marked inhibition of cell proliferation of both cell lines with the two-agent regimen compared to the one-agent regimen. Asterisks indicated the statistical significance between multi-group comparisons. (**C**) Western blotting showing the levels of PKM2 and PKM1 in T24 cells treated with DMSO, cisplatin (1 μg/ml), shikonin (1 μM) or both. Note the comparable levels of the proteins in different treatment groups. (**D**) Western blotting of treated T24 cells showing the upregulated levels of cleaved PARP and BiP (GRP78) in cisplatin-treated cells; cleaved caspase 3, cleaved caspase 7 and LC3-I in shikonin-treated cells; and all of these proteins in the dual-agent treatment group. The blot images were cropped to save space and their full-length versions are available upon request.

To assess whether the status of PKM2 expression affected how BC cells responded to conventional chemotherapeutic, cisplatin, we compared the proliferation capability of PKM2-expressing T24 cells with PKM2-knockdown T24 cells (Fig. 2A) in the presence of 1 μg/ml cisplatin. PKM2-knockdown T24 cells exhibited significantly greater inhibition of proliferation (Fig. 2B) and higher degree of apoptosis (Fig. 2C) than PKM2-expressing T24 cells. Additionally we found that, in a dual-agent regimen, cisplatin and shikonin together exhibited greater inhibitor effects on both T24 and RT112 cell lines, compared with single-agent treatments (Fig. 3A,B). For T24 cells, cisplatin and shikonin achieved an 80% inhibition of cell proliferation, compared to 30 and 50%, respectively, when used alone (Fig. 3A). For RT112 cells, cisplatin and shikonin together achieved an 80% inhibition of cell proliferation, compared to 20 and 40%, respectively, when used alone (Fig. 3B). The calculated combination index^21^ in the dual-agent regimen for T24 and RT112 was 0.9 and 0.6, respectively, indicating “slight synergism” and “synergism”, respectively, for the two cell lines. Western blotting analyses showed that single and dual-agent treatments did not significantly affect the levels of PKM2 and PKM1 per se (Fig. 3C). In contrast, cisplatin upregulated apoptotic marker cleaved PARP and unfolded protein response protein BiP, whereas shikonin upregulated apoptotic marker cleaved caspase 3 and 7 and autophagy marker LC3-I. Other markers such as LC3-II and Atg7 were unaffected. Induction of different players in these pathways by shikonin and cisplatin might explain at least in part the synergism between these two agents.

### Acquired Chemoresistance of BC Cells Is associated with PKM2 Overexpression

**Figure 4:**
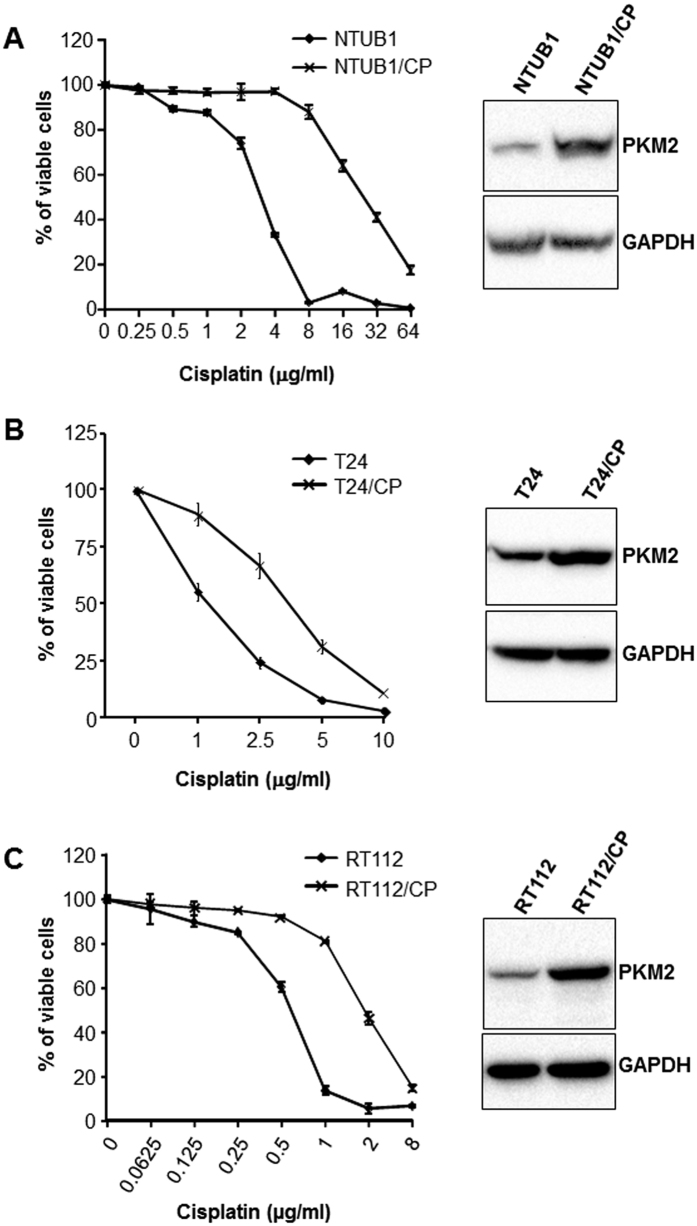
Acquired cisplatin-resistance in BC cells is associated with an upregulation of PKM2. BC cell lines NTUB1 (**A**), T24 (**B**) and RT112 (**C**) were passaged in media containing escalating concentrations of cisplatin as indicated. Both parental cell lines and cisplatin-resistant cell lines (indicated as/CP) obtained from the various concentrations of cisplatin were then assessed for their proliferation status by WST-1 assay (left panels of (**A**–**C**)) and the levels of PKM2 by Western blotting (right panels of (**A**–**C**)) at the end of 72 hours of subculture at 1 μg/ml cisplatin concentration point for all the three cell lines. Note the generation of cisplatin-resistant cell lines that were more viable in high concentrations of cisplatin for each of the three parental cell lines and the markedly increased PKM2 expression in these cisplatin-resistant cells compared to parental cells. The blot images were cropped to save space and their full-length versions are available upon request.

Because most advanced BCs in humans eventually develop resistance to cisplatin^4^,^8^,^9^, we explored whether PKM2 played a role in this process. We performed serial passages of NTUB1, T24 and RT112 cell lines in the presence of increasing concentrations of cisplatin (Fig. 4A–C, left panels). Although the extent of resistance to cisplatin varied among the three cell lines, all of them acquired some degree of resistance, e.g., being capable of continued growth in at least 1 μg/ml cisplatin. Without exception, the cisplatin-resistant cells had marked upregulation of PKM2, compared to their parental counterparts, as evidenced by Western blotting (Fig. 4A–C, right panels). In a separate set of experiments, we found that these cisplatin-resistant cells were highly sensitive to shikonin treatment, leading to significant levels of growth inhibition (Supplemental Fig. 1).

### Shikonin Suppresses Actin-polymerization and BC Cell Migration and Invasion

**Figure 5:**
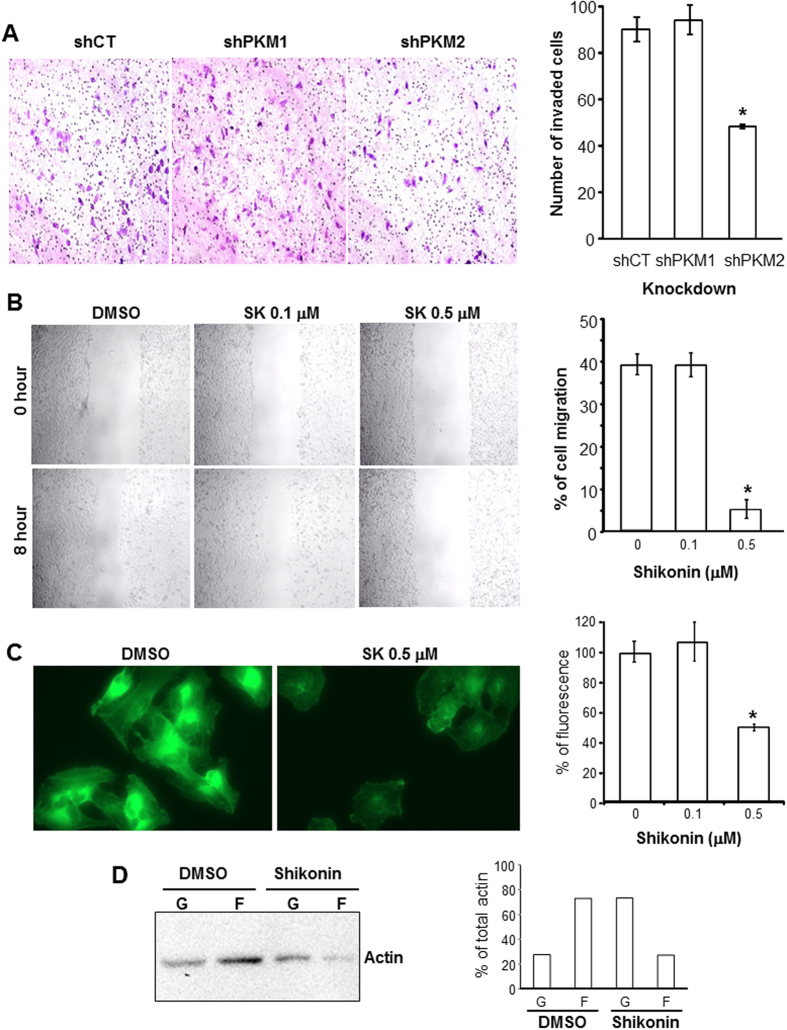
Inhibition of PKM2 by shRNA or shikonin leads to reduced BC cell migration and invasion. (**A**) T24 cells that had stably incorporated shCT, shPKM1 and shPKM2 were subjected to Matrigel invasion assay. Invaded cells were stained with H&E (left three panels) and counted and expressed as the number of invaded cells (right panel). Note that the knockdown of PKM2 but not PKM1 significantly reduced cell invasion. All experiments were done in triplicates. (**B**) Wound-healing/cell migration assay of T24 cells in the presence of DMSO, 0.1 μM and 0.5 μM of shikonin. After cultured T24 cells reached 90% confluence, a scratch wound was introduced with a sterile pipet tip and phase-contrast images were recorded at 200 × magnification at 0 and 8 hours post-scratching, and the distances between the leading edges of cell migration at 8 h post-wounding were measured, averaged and referenced to that of 0 h group and expressed as percentage of cell migration. Note the significantly decreased cell migration in the presence of 0.5 μM shikonin. (**C**) Assessment of actin polymerization in T24 cells treated with DMSO or 0.5 μM shikonin. Permeabilized T24 cells were stained with ActinGreen-488 and the stained cells were either subjected to fluorescent microscopy (left two panels at 200 x) or to fluorescence quantification by spectrometry at 495 nm, with the reading normalized against the control cells set as 100%. Note the marked decrease of actin polymerization in shikonin-treated cells. (**D**) Biochemical assay of G-actin (**G**) and F-actin (**F**). DMSO- and shikonin (0.5 μM)-treated T24 cells were subject to the quantification of G- and F-actin using a commercial kit (see Methods). Left panel was Western blotting showing the relative abundance of G- and F-actin in equal amounts of starting material, and the right panel was the semi-quantification of the bands in the left panel, expressed as the percentage of G- or F-actin in the total actin. Note the significant reduction of F-actin and increase of G-actin in shikonin-treated cells. The blot images were cropped to save space and their full-length versions are available upon request.

To examine whether, in addition to the inhibitory effects on cell proliferation, down-regulation of PKM2 by shRNA or treatment of cells with shikonin also could affect other processes in tumor progression, we performed cell invasion assay on parental T24 cells and T24 cells deficient for PKM1 or PKM2 (Fig. 5A). We observed that the extent of invasion was significantly diminished in PKM2-deficient T24 cells than in PKM1-deficient cells and parental T24 cells (Fig. 5A). Similarly, shikonin treatment of parental T24 cells led to an 8-fold reduction in cell migration (Fig. 5B). Shikonin-treated T24 cells exhibited a marked decrease of actin polymerization (Fig. 5C) and F-actin/G-actin ratio (Fig. 5D), compared to the un-treated controls. Together, these results indicate another important mechanism whereby shikonin inhibits tumor progression.

### Combination Therapy with Shikonin and Cisplatin Inhibits BC Growth and Metastases *In Vivo*

To determine whether shikonin could also inhibit mouse BC cell proliferation, thus setting a stage for syngeneic model experiments, we exposed MBT2 and MB49 mouse BC cell lines both of which expressed PKM2 (Supplemental Fig. 2A) to cisplatin, shikonin, or both. As with the human cell lines, a dose-dependent inhibition of cell proliferation was observed with either agent (Supplemental Fig. 2B and 2C). In combination therapies, MB49 cells responded more sensitively to cisplatin and shikonin than MBT2 cells (Supplemental Fig. 2D) and were chosen for *in vivo* experiments.

**Figure 6:**
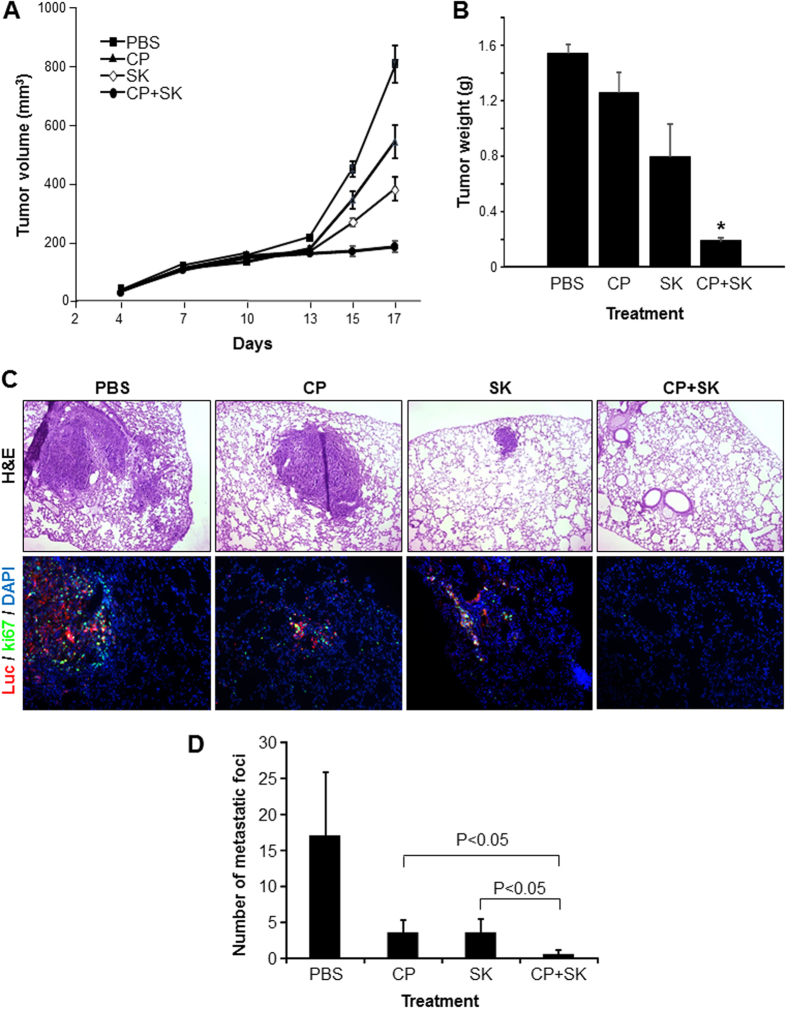
Synergistic effects of cisplatin and shikonin on BC growth and metastasis *in vivo*. (**A** and **B**) Cultured MB49 mouse BC cells (2 × 10^6^ in 100 μl PBS) were injected subcutaneously into the rear back of 12 week-old, C57BL/6 male mice. Tumor sizes were measured at the indicated time points using a Vernier caliper. On day 7 when tumors reached 100 mm^3^, tumor-bearing mice (N = 5 per treatment group) received intraperitoneal injection of PBS, cisplatin (3 mg/kg), shikonin (2 mg/kg) or both cisplatin and shikonin. Additional injections were performed on day 10, 13 and 15. All the mice were sacrificed on day 17. The tumor volumes were calculated by the formula V = (L*W*W)/2 and presented as means ± SEM (**A**). Tumor weights were measured at the end point of the excised tumors (**B**). Note the dramatic reduction of tumor sizes and weights in the dual-agent treatment group, compared to un-treated and single-agent treatment groups. (**C** and **D**) Detection and quantification of lung metastases. Lung metastases were detected by surveying the H&E-stained lung tissues (**C**, upper panel) and by double immunofluorescence labeling of firefly luciferase and ki67 (**C**, lower panel). (**D**) The metastatic foci per mouse were counted, averaged and presented as means ± SEM (see Materials and Methods for details). Note the marked reduction of lung metastasis in dual-agent group compared to un-treated control group and single-agent groups.

After cultured MB49 cells were implanted subcutaneously into syngeneic mice and were allowed to grow into 100 mm^3^ tumor mass, the mice were treated with PBS, cisplatin, shikonin or cisplatin plus shikonin on fixed schedules (see Materials and Methods). We observed striking differences in tumor growth at different times (Fig. 6A) and more so at the time of sacrifice (Fig. 6B). While cisplatin and shikonin as single agents inhibited tumor volume and size by about 20 and 50%, respectively, these two agents together led to more than 80% tumor inhibition (Fig. 6A,B). A marked reduction of lung metastases was also evident in the two-agent group compared to the single-agent groups (Fig. 6C,D). These results are strongly supportive of synergistic effects of cisplatin and shikonin in significantly curtailing BC growth and progression.

## Discussion

Our present study provided significant amounts of experimental evidence advancing the understanding of PKM2 and BC, a highly prevalent, challenging, but historically under-investigated cancer. First and foremost, despite the increasingly appreciated role of PKM2 in oncogenesis in several non-BC types^22^,^23^, the association of PKM2 with BC was only mentioned occasionally in the literature and not significantly beyond the conflicting results of whether plasma concentrations of PKM2 were diagnostic of urological malignancies^24^,^25^. A rare exception was the attempt to determine, by mass spectrometry, the relative abundance of PKM2 versus its alternatively spliced isoform PKM1^26^. The latter study employed four “human urothelial carcinoma specimens” with no reference to grade or stage and “two normal bladder controls” with no information on whether they were urothelial or non-urothelial tissues. The authors concluded that there was no evidence of a shift from PKM1 to PKM2 expression in tumor tissues^26^, thus arguing against a role of PKM2 in bladder tumorigenesis. In a recent paper, however, we provided differing data showing marked overexpression of PKM2 during the initiation of low-grade non-invasive bladder tumors in transgenic mice expressing a constitutively activated HRAS in the urothelium^19^. We subsequently extended this finding of PKM2 overexpression to high-grade muscle-invasive tumors in hybrid mice expressing activated HRAS and lacking p53, human cell lines representing low-and high-grade BCs and human BC specimens of both low- and high grades^19^. Our data are supported strongly and independently by the TCGA consortium using RNA-sequencing that identified 97% PKM2 transcripts and only 3% PKM1 transcripts in 131 cases of muscle-invasive human BCs^20^. Thus, data from *in vitro* and *in vivo* and mouse and human all illustrate that PKM2 is preferentially selected over PKM1 in early and late-stage bladder tumor cells. By demonstrating that down-regulation or chemical inhibition of the oncogenic activity of PKM2 markedly decreases BC cell proliferation (Figs 1, 2, 3 and Supplemental Fig. 2), cell migration and invasion *in vitro* (Fig. 5) and reduces bladder tumor growth and metastases *in vivo* (Fig. 6), our present study lends further support on a functional level to the emerging concept that PKM2 is important for bladder tumorigenesis and progression. It should be noted that linking PKM2 inhibition to reduced actin polymerization (Fig. 5C,D) *in vitro* and tumor metastases *in vivo* (Fig. 6) had not been shown before. Whether such effects can be extended to non-BC cells and the detailed cellular mechanism(s) underlying these effects warrant further investigation.

The second significant aspect of our present study is the experimental evidence we generated supporting and extending PKM2 as a major target of shikonin. Shikonin is a chemical found in the roots of medicinal plants such as *Lithospermum erythrorhizon* that has been used empirically to treat inflammatory and infectious diseases^27^,^28^. The chemical structure places it in the naphthoquinone category^29^. We showed that shikonin binds PKM2 with a reasonable level of specificity because it does not bind PKM1, MAPK or AKT in the identical conditions as evidenced by the lack of these proteins upon pull-down/Western blotting (Fig. 1A,B). Our result is therefore in line with those initially obtained from non-BC cells^17^, and suggests that the shikonin-PKM2 interaction is operative in BC cells. We also showed that shikonin, like the down-regulation of PKM2 by RNAi, inhibits BC cell proliferation and, more importantly, that BC cells stably expressing the shRNA for PKM2 were less sensitive to shikonin inhibition than the control cells constitutively overexpressing PKM2 (Fig. 1D). We further showed that, at the concentration that shikonin is inhibitory of cell proliferation, it does not affect PKM2′s pyruvate kinase activity (Fig. 1E), suggesting specific role(s) of shikonin in inhibiting oncogenesis. We are fully cognizant of the fact that almost all chemicals have off-target effects. By suggesting a role of shikonin in inhibiting PKM2, we are in no way excluding the possibility that shikonin also acts on other targets, some of which have already been identified^30^. However, we believe that it is the totality of the cellular effects, rather than the potential off-target effects, of a potential cancer drug that should be the justification for in-depth exploration (see below).

The third significant aspect of our present study is our first demonstration that PKM2 overexpression is associated with the resistance of BC cells to cisplatin. We found that the continuous passages of three different cell lines (e.g., NTUB1, T24 and RT112) in increasing concentrations of cisplatin confers resistance to this commonly used chemotherapeutic and that PKM2 is consistently upregulated in all the resultant resistant cell lines (Fig. 4). We found that the cell lines expressing less PKM2 (e.g., by shRNA down-regulation) were more sensitive to cisplatin in terms of reduced proliferation and increased apoptosis than those expressing more PKM2 (Fig. 2). We also found that shikonin significantly potentiates the inhibition of cisplatin on all the BC cell lines tested, via non-redundant induction of several apoptotic and autophagic promoters. Furthermore, we found that experimentally induced cisplatin-resistant cells that overexpressed PKM2 responded sensitively to shikonin (Supplemental Fig. 1). Finally, shikonin and cisplatin together, but not used as single agents, dramatically reduced tumor growth and metastasis of a highly aggressive mouse BC cell line, MB49 (Fig. 6). These results raise the interesting prospect of using shikonin as a potential therapeutic to reduce or even prevent the intrinsic as well as acquired chemoresistance to cisplatin or as an adjunct in standard chemo- and/or radiotherapy. The differences in the sensitivity of MB49 to shikonin and cisplatin treatment between *in vitro* (Supplemental Fig. 2D) and *in vivo* (Fig. 6A,B) models may reflect the potential treatment effects on the tumor microenvironment which is absent in cultured cells. It may also be due to the nutrition-rich conditions of the cultured cells which may render the treatment less effective. Studies are underway to generate cisplatin-resistant mouse BC cell lines (e.g., MB49) that will allow us to evaluate the inhibitory effects of shikonin in additional syngeneic models. Since PKM2 is overexpressed in low-grade non-invasive (pTaG1), high-grade non-invasive (pTaG2/3) and high-grade invasive BCs (pT1-4)^19^,^20^, its inhibition should benefit most BC variants. Further studies including those testing the toxicity profile, delivery routes (systematic vs. intravesicle), vehicles (siRNA of PKM2 in nanoparticles) and bioavailability are required to advance our laboratory findings to the bedside.

## Materials and Methods

### Bladder cancer cell lines and culture conditions

The T24 cell line, originally cultivated from an 81-year old woman bearing a recurrent grade-3 bladder cancer (BC)^31^, was purchased from American Type Culture Collection (ATCC; Manassas, VA). The RT112 cell line, originally derived from a 63-year old man with a primary grade-2 BC^32^, was acquired as a gift from Dr. Margaret Knowles of Cancer Research UK Clinical Center. The NTUB1 cell line, originally derived from a 70-year-old female patient with a high-grade BC^33^ was a kind gift of Dr. Kuo-How Huang of National Taiwan University, Taiwan. The MB49, a dimethylbenzanthracene (DMBA)-transformed cell line from the urinary bladders of C57BL/6 mice^34^, was a generous gift from Dr. Yi Luo of University of Iowa. The cell line had been engineered to stably express a luciferase reporter gene to facilitate detection in animal models^35^. The MBT2 cell line, originally derived from a poorly differentiated bladder tumor induced by chemical carcinogen N-[4-(5-nitro-2-furyl)-2-thiazolyl] formamide in C3H/He mice^36^, was obtained from Dr. Ian Mohr of New York University School of Medicine. All the 5 cell lines used in this study were rapidly proliferating with a population doubling time of less than 24 h; exhibited invasive properties *in vitro*; and were tumor-forming in nude mice. The T24 and MBT2 cells were cultured in DMEM containing 10% fetal bovine serum. The RT112, NTUB1 and MB49 cells were grown in RPMI 1640 medium supplemented with 10% fetal bovine serum. All cell culture reagents were purchased from GIBCO - Thermo Fisher Scientific (Grand Island, NY).

### Knockdown of PKM2 by shRNA

The T24 human BC cell line was chosen for the knockdown of mRNA encoding PKM2 with that encoding PKM1 as a control using specific shRNAs (designated as shPKM2 and shPKM1) that we described previously^19^. Non-specific, scrambled shRNA was used as a negative control (shCT). The shPKM2 and shPKM1 were cloned into pLKO.1-TRC and packaged with plasmids psPAX2 and pMD2.G (all the three plasmids from Addgene, Cambridge, MA) in HEK 293 T cells. The lentiviral preparations were then used to transduce the T24 cells in presence of 8 μg/mL polybrene. The transduced cells were selected in 1 μg/ml puromycin for stable clones, and the specificity and efficiency of the knockdown were verified by RT-PCR followed by Western blotting using antibodies specific for PKM2 and PKM1 (see later).

### Pull-down of shikonin-interacting proteins

Total proteins were extracted from 95% confluent T24 cells stably transduced with shCT or shPKM2 in a lysis buffer (20 mM Tris-HCl/pH 7.5, 150 mM NaCl, 1 mM EDTA, 1 mM EGTA, 1% Triton, 2.5 mM sodium pyrophosphate, 1 mM beta-glycerophosphate, 1 mM Na3VO4, plus a cocktail of proteinase inhibitors (Thermo Fisher Scientific, Waltham, MA)). The soluble proteins were loaded onto solid-phase shikonin (Cayman Chemical, Ann Arbor, MI) according to a published procedure^17^. The mixture was incubated with constant rotation at 4 °C for 2 h and then centrifuged at 5,000 × g at 4 °C for 2 min. After washing 3 times in a washing buffer (50 mM Tris–HCl/pH 7.5, 150 mM NaCl, 2 mM EDTA, 10% glycerol and 1 mM NaF), the bound proteins were eluted in SDS-sample buffer at 95 °C for 5 min and then resolved on 12% SDS-PAGE and stained with silver nitrate or immuno-blotted with various antibodies (see later).

### Pyruvate kinase activity assay

The pyruvate kinase activity of shikonin-treated T24 and RT112 cells was measured using a kit from Sigma-Aldrich (St Louis, MO). Briefly, 95% confluent cells were lysed in the assay buffer and centrifuged at 13,000 × g at 4 °C for 10 min. The supernatants were transferred into a master reaction mix and incubated at room temperature for 30 min in the dark, after which they were read by spectrometry at 570 nm. Values were determined by plotting against a standard curve prepared using standards included in the kit. All samples were in triplicate.

### Treatment of cultured BC cell lines with shikonin and/or cisplatin

Shikonin and cisplatin were obtained from Cayman Chemical (Ann Arbor, MI) and US National Cancer Institute’s Developmental Therapeutics Program. Both chemicals were made into stock solutions (10 mM of shikonin in DMSO and 1 mg/ml cisplatin in 0.9% NaCl) before being serially diluted in corresponding culture media. After incubation for various lengths of time indicated in different figures, the remaining cells were harvested and subjected to a range of assays describe in other sections. The drug combination index was calculated using CompuSyn software (ComboSyn, Inc, Paramus, NJ) described by Chou and colleagues^21^.

### Determination of cell proliferation and apoptosis

The cell proliferation rate was determined using the WST-1 assay kit (Clontech, Mountain view, CA). Briefly, the cultured cells were seeded at a density of 2,000 cells per well in 96-well plates and treated with the various agents and conditions described under other parts of this section. At the end of the treatments, WST-1 reagents were added and incubated for 2 h before the plates were read by spectrometry at 450 nm. All samples were in triplicate. Apoptosis was measured by staining cultured cells with an Annexin V-FITC apoptosis staining kit (Cell Signaling), followed by fluorescence-activated cell sorting.

### Generation of cisplatin-resistant cells

The cisplatin-resistant cell lines for T24 (T24/CP) and RT112 (RT112/CP) were generated from non-resistant parental cells by serially passaging in culture medium containing escalating concentrations of cisplatin from 0.1 μg/ml to 1 μg/ml. The resistant cells had been maintained in 1 μg/ml cisplatin for 6 months before analysis. The cisplatin-resistant NTUB1/CP cell line had been selected in the culture medium containing 12 μM cisplatin and maintained in the medium containing 2 μg/ml cisplatin (~6.6 μM).

### *In vitro* cell migration and invasion assays

The T24 cell line was chosen for both cell migration and invasion assays. For the cell migration assay, T24 cells (2 × 10^5^/well) were seeded onto 24-well plates and, when the cells reached 90% confluence, the wounds were introduced with a sterile pipette tip. The cells were continuously cultured in the medium containing DMSO or that containing 0.1 μM or 0.5 μM shikonin. The extent of cell migration was recorded by phase contrast microscopy at 0 and 8 h post-wounding. The average distance between the leading edges of cell migration at 8 h was referenced to 0 h within each treatment group and expressed as the percentage of cell migration.

For the cell invasion assay, BioCoat Matrigel Invasion Chamber (BD Biosciences, San Jose, CA) was used. The T24 cells (2.0 × 10^4^ cells) stably expressing shCT, shPKM1 and shPKM2 were seeded into the upper chamber after the reconstitution of the cells with Matrigel. The cells were cultured for 72 h and then treated with 20 ng/ml 12-O-tetradecanoylphorbol-13-acetate, a chemo-attractant to stimulate cell invasion. The non-invading cells in the upper chamber were removed by Q-tips and the invaded cells underneath the membrane were visualized by H&E staining and counted in 5 fields (1-center and 4-peripheral) under 200 × magnification for each well and the numbers from the triplicates were averaged.

### Syngeneic mouse model

Twelve-week old male C57BL/6 mice (Jackson Lab, Bar Harbor, ME), the same strain from which the MB49 cell line was originally derived^35^, were used as recipient mice. After acclimatization, the mice were randomized into 4 groups (5 per group) and injected subcutaneously in the rear back with cultured MB49 cells (2 × 10^6^ in 100 μl PBS). When the tumors grew to 100 mm^3^ on day 7, the 4 groups of mice were injected intraperitoneally with each of the following: (i) PBS, (ii) cisplatin (3 mg/kg in PBS), (iii) shikonin (2 mg/kg in DMSO then reconstituted in PBS), and (iv) 3 mg/kg cisplatin in PBS plus 2 mg/kg shikonin in DMSO then reconstituted in PBS. The injections were repeated on day 10, 13 and 15 after tumor cell injection. The tumor sizes were measured using a Vernier caliper on days 4, 7, 10, 13, 15 and 17 and the tumor volumes were calculated with formula V = (L*W*W)/2 and presented as means ± SEM. All the mice were sacrificed on day 17 and the tumors were weighed. The lung tissues were also procured at the time of sacrifice to assess the extent of metastasis. They were fixed in PBS-buffered 10% formalin, paraffin-embedded and cut into 5 μm thick sections for either H&E or immunofluorescence staining (see later). For the quantification of metastastic foci, 30 constitutive sections were cut from the lung tissue per mouse. One of every 5 sections was H&E-stained and all the metastatic loci on each section were counted at 400 × magnification, and the total foci per 6 sections per mouse were presented with standard deviation. The animal-related work was conducted in accordance with the federal regulations and after the approval of a protocol by the Institutional Animal Care and Use Committee of New York University School of Medicine.

### Western blotting and immunofluorescent staining

Total proteins from various cell lines before and after experimental inventions described under other parts of this section were extracted with 2 × SDS-sample buffer, resolved on 12% SDS-PAGE (acrylamide:bisacrylamide ratio 30:1), electrophoretically transferred onto Immobilon-PVDF membrane and blotted with the primary antibodies against PKM2, MAPK, AKT, cleaved-PARP, cleaved-caspase 3, cleaved-caspase 7, LC3, BiP (GRP78), IRE1, Atg7 (all from Cell Signaling, Denvers, MA; all with 1:1,000 dilution except anti-MAPK which was diluted 1:2,000), and PKM1 (Sigma-Aldrich, St. Louis, MO; 1:1,000). For immunofluorescence staining of F-actin in cultured T24 cell with or without shikonin treatment, live cells were prefixed in 3.7% formaldehyde for 10 min and then permeabilized in 0.1% Triton X-100 in PBS for 5 min, followed by staining with ActinGreen-488 (GeneCopoeia, Rockville, MD). For quantification of F-actin, the fluorescence of independent batches of ActinGreen-488-stained cells was measured *in situ* by spectrophotometry, and the result was normalized against the control cells set as 100%. For immunofluorescence detection of MB49 cells metastasized to the lungs, their paraffin-sections were subjected to antigen unmasking by microwaving in a citrate buffer (pH 6.0) for 20 min and then to double staining using primary antibodies against firefly-luciferase (Promega, Madison, WI; 1:400) and ki67 (1:400; Cell Signaling) and secondary antibodies conjugated with alexa-488 or alexa-594.

### Biochemical quantification of F-actin and G-actin

The relative abundance of F-actin and G-actin with or without shikonin treatment was determined using an *In Vivo* Assay Biochem Kit (Cytoskeleton, Inc., Denver, CO). Briefly, the T24 cells were exposed to either DMSO or that containing 0.5 μM shikonin for 6 h. The cells were then lysed in LAS2 buffer and the lysate was centrifuged at 350 × g for 5 min at room temperature. The supernatant was centrifuged at 100,000 × g at 37 °C for 1 h to separate F-actin from G-actin. The pellet containing F-actin was re-suspended in actin de-polymerization buffer on ice for 1 h. Equal volumes of G-actin supernatant and F-actin resuspension were analyzed by Western-blotting and the actin bands were semi-quantified using NIH ImageJ software.

### Statistical analysis

The statistical analysis was done with Prism 6 (GraphPad Software, La Jolla, CA). Student’s t tests (two-tailed) were used to calculate the statistical significances with a P value of <0.05 considered significant.

## Additional Information

**How to cite this article**: Wang, X. *et al*. Inhibition of Pyruvate Kinase M2 Markedly Reduces Chemoresistance of Advanced Bladder Cancer to Cisplatin. *Sci. Rep.* **7**, 45983; doi: 10.1038/srep45983 (2017).

**Publisher's note:** Springer Nature remains neutral with regard to jurisdictional claims in published maps and institutional affiliations.

## Supplementary Material

Supplemental Information
