# Supplementary material for: Inhibition of Pyruvate Kinase M2 Markedly Reduces Chemoresistance of Advanced Bladder Cancer to Cisplatin
Source: Sci Rep. 2017 Apr 5;7:45983. doi: 10.1038/srep45983 (PMC5380992; doi:10.1038/srep45983)
Supplement: Supplemental Information [file srep45983-s1.pdf]

Inhibition of Pyruvate Kinase M2 Markedly Reduces Chemoresistance of Advanced Bladder Cancer to Cisplatin

Xing Wang, Fenglin Zhang and Xue-Ru Wu

Supplemental Figure 1. Cisplatin-resistant BC cell lines respond sensitively to shikonin treatment. Parental and cisplatin-resistant T24 (A) and RT112 (B) BC cells were cultured in the media containing 0, 0.25 and 0.5 μM of shikonin for 72 hours and then subjected WST-1 cell proliferation assay. Asterisks denote statistical significance between cisplatin-sensitive and resistant cell lines. Note that cisplatin-resistant T24/CP and RT112/CP responded more sensitively to shikonin than their parental counterparts, e.g., T24 and RT112.

Supplemental Figure 2. Combined effects of cisplatin and shikonin on mouse BC cell lines MBT2 and MB49. (A) Western blotting showing that both MBT2 and MB49 expressed PKM2. The blot images were cropped to save space and their full-length versions are available upon request. (B) Determination of IC50 of MBT2 or MB49 mouse BC cell lines to cisplatin and shikonin. The two cell lines were seeded in 96-well plates and incubated with cisplatin (B) or shikinon (C) at the concentrations indicated for 72 hours, after which they were subjected to WST-1 assay. Each condition was done in triplicates and the cell proliferation values were normalized to the mock group. (D) MBT2 and MB49 cells were incubated with PBS, cisplatin (0.5 μg/ml for MBT2 and 0.4 μg/ml for MB49), shikonin (0.25 μM for MBT2 and 0.5 μM for MB49) or both. After 72 hours, WST-1 was performed to assess proliferation. Note that cisplatin and shikonin together had more inhibition on proliferation than either agent alone particularly for the MB49 cells.

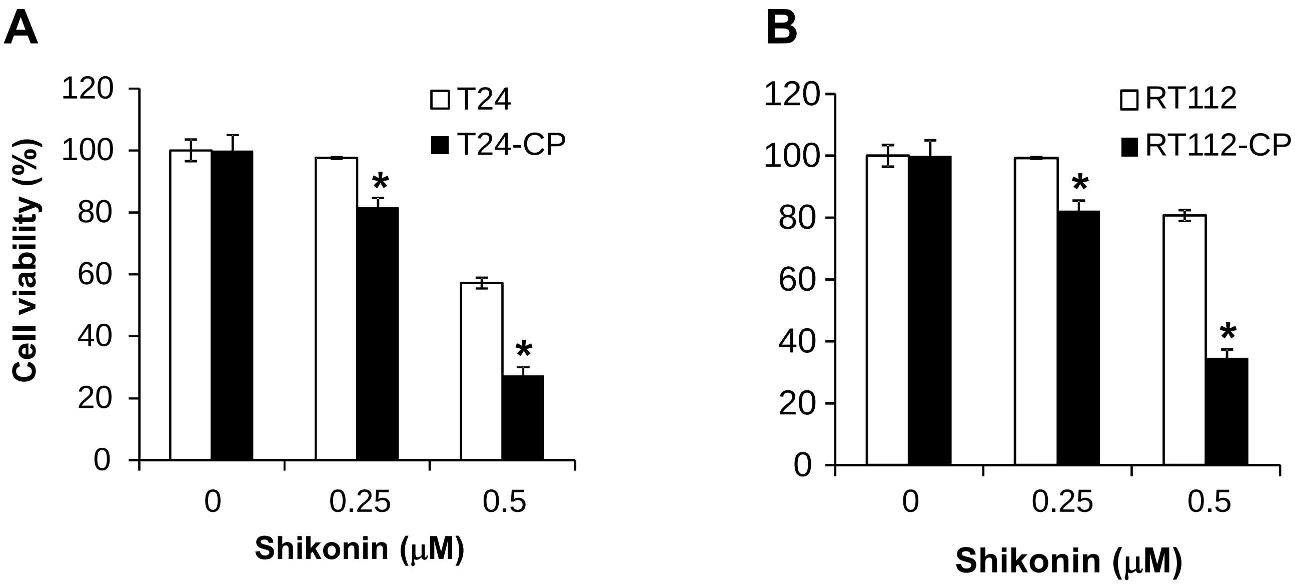

Supplemental Fig. 1

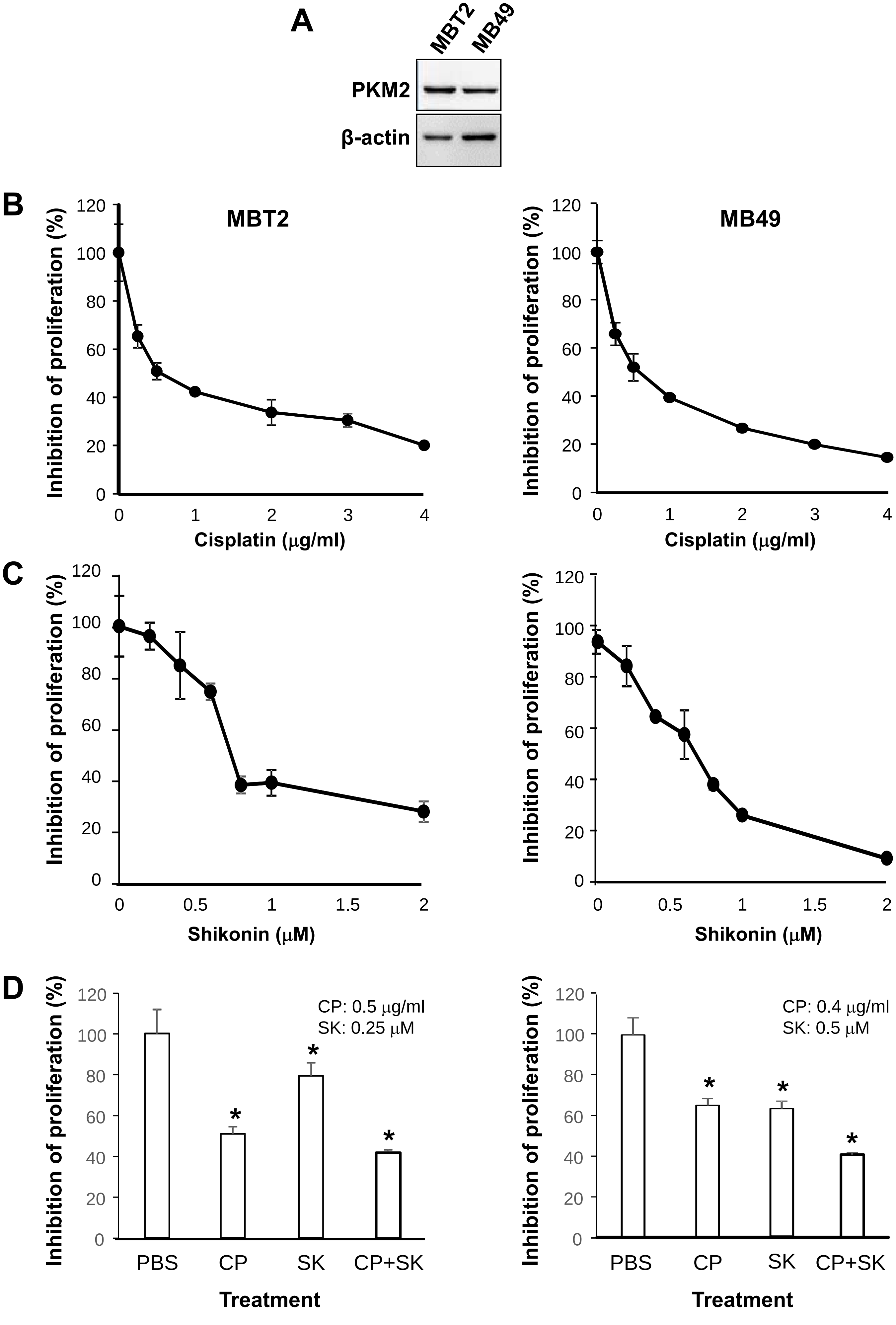

Supplemental Fig. 2
